# Predictive utility of ANCA positivity and antigen specificity in the assessment of kidney disease in paediatric-onset small vessel vasculitis

**DOI:** 10.1136/rmdopen-2024-004315

**Published:** 2024-06-17

**Authors:** Simranpreet K Mann, Jeffrey N Bone, Else S Bosman, David A Cabral, Kimberly A Morishita, Kelly L Brown

**Affiliations:** 1 Department of Microbiology and Immunology, Faculty of Science, University of British Columbia, Vancouver, British Columbia, Canada; 2 British Columbia Children's Hospital Research Institute, Vancouver, British Columbia, Canada; 3 Division of Rheumatology, Department of Pediatrics, Faculty of Medicine, University of British Columbia, Vancouver, British Columbia, Canada; 4 British Columbia Children's Hospital, Vancouver, British Columbia, Canada

**Keywords:** Vasculitis, Autoantibodies, Autoimmune Diseases, Child, Autoimmunity

## Abstract

**Objectives:**

The objective of this study is to evaluate whether anti-neutrophil cytoplasmic antibody (ANCA) seropositivity and antigen specificity at diagnosis have predictive utility in paediatric-onset small vessel vasculitis.

**Methods:**

Children and adolescents with small vessel vasculitis (n=406) stratified according to the absence (n=41) or presence of ANCA for myeloperoxidase (MPO) (n=129) and proteinase-3 (PR3) (n=236) were compared for overall and kidney-specific disease activity at diagnosis and outcomes between 1 and 2 years using retrospective clinical data from the ARChiVe/Paediatric Vasculitis Initiative registry to fit generalised linear models.

**Results:**

Overall disease activity at diagnosis was higher in PR3-ANCA and MPO-ANCA-seropositive individuals compared with ANCA-negative vasculitis. By 1 year, there were no significant differences, based on ANCA positivity or specificity, in the likelihood of achieving inactive disease (~68%), experiencing improvement (≥87%) or acquiring damage (~58%). Similarly, and in contrast to adult-onset ANCA-associated vasculitis, there were no significant differences in the likelihood of having a relapse (~11%) between 1 and 2 years after diagnosis. Relative to PR3-ANCA, MPO-ANCA seropositivity was associated with a higher likelihood of kidney involvement (OR 2.4, 95% CI 1.3 to 4.7, p=0.008) and severe kidney dysfunction (Kidney Disease Improving Global Outcomes (KDIGO) stages 4–5; OR 6.04, 95% CI 2.77 to 13.57, p<0.001) at onset. Nonetheless, MPO-ANCA seropositive individuals were more likely to demonstrate improvement in kidney function (improved KDIGO category) within 1 year of diagnosis than PR3-ANCA seropositive individuals with similarly severe kidney disease at onset (p<0.001).

**Conclusions:**

The results of this study suggest important paediatric-specific differences in the predictive value of ANCA compared with adult patients that should be considered when making treatment decisions in this population.

**WHAT IS ALREADY KNOWN ON THIS TOPIC**

- In adults with anti-neutrophil cytoplasmic antibody (ANCA)-associated vasculitis (AAV), ANCA specificity towards proteinase-3 and myeloperoxidase have been identified as predictive biomarkers for severe disease, kidney involvement and relapse.
- It is unclear whether ANCA positivity and specificity have more, less or equal predictive utility in paediatric-onset vasculitis.

**WHAT THIS STUDY ADDS**

- This study uses clinical data collected over a period of 15+ years from >400 children and adolescents with small vessel vasculitis to evaluate the predictive utility of ANCA.
- For paediatric patients with kidney disease, ANCA positivity and specificity have prognostic utility for assessing outcomes 1 year after diagnosis.
- ANCA positivity and specificity are not associated with an increased likelihood of achieving inactive disease, experiencing improvements in disease, or accruing damage at 1-year postdiagnosis, or experiencing a relapse between 1-year and 2-year postdiagnosis.

**HOW THIS STUDY MIGHT AFFECT RESEARCH, PRACTICE OR POLICY**

- Results from this study show that the utility of ANCA as a predictive biomarker in paediatric-onset AAV differs from their reported use in adult-onset AAV and should be taken into consideration when devising treatment plans for children and adolescents with AAV.

## Introduction

Anti-neutrophil cytoplasmic antibody (ANCA)-associated vasculitis (AAV) describes a group of rare vasculitides that, for the majority of patients, are associated with the presence of ANCA against one of two (auto)antigens: proteinase-3 (PR3-ANCA) or myeloperoxidase (MPO-ANCA).^1^ AAV comprises granulomatosis with polyangiitis (GPA), microscopic polyangiitis (MPA) and eosinophilic GPA (EGPA), with PR3-ANCA and MPO-ANCA frequently associated, respectively, with GPA and MPA.

Prior to the current treatment era and the use of cyclophosphamide or rituximab, plus glucocorticoids, most paediatric AAV patients died within a year of diagnosis, usually due to lung or kidney failure, or infection. Although current treatments are life-saving, they are not curative, thus, AAV is now a chronic disease.^2 3^ The current therapeutic paradigm is to treat aggressively enough to prevent irreversible (vasculitis-induced) damage yet limit medication-associated toxicity resulting from the ‘over-treatment’ of disease that is mild, or no longer active.

Compared with adult-onset AAV, AAV in children and adolescents is more likely to be severe at outset and more likely to present with (moderate-to-severe) multiorgan involvement.^4–6^ Our previous studies have shown that nearly 80% of cases of paediatric AAV have kidney disease at onset, and within 1 year of diagnosis, 70% have abnormal kidney function and 35% have permanent kidney damage.^7^ The presence of kidney disease in paediatric-onset AAV is a major driver of treatment decisions. There is an argument for optimising treatment precision to potentially avoid the continuous accrual of damage from both the disease and treatment toxicity over the course of a young person’s life.

Because of the rarity of paediatric-onset AAV, most knowledge about the disease is obtained from the study of adult-onset AAV. Studies in adults have demonstrated that the presence and specificity of ANCA (for PR3 or MPO) can be used as biomarkers to predict disease severity, outcomes and risk for relapse.^8 9^ PR3-ANCA is associated with multiorgan involvement, frequently in the lungs,^8 10 11^ and a higher rate of relapse^8^ that is more responsive to treatment with rituximab rather than cyclophosphamide.^12 13^ In contrast, MPO-ANCA-positive AAV in adults typically presents with kidney-limited disease that is frequently more severe at presentation and more likely to progress to kidney failure^8 10 11^; for these individuals, cyclophosphamide may be preferred for remission-induction instead of rituximab.^14^

The present study aimed to evaluate the potential clinical utility of ANCA positivity and specificity (for PR3 or MPO) as prognostic markers in paediatric-onset small vessel vasculitis, inclusive of AAV, and, of particular interest, whether our findings suggest similar, improved or limited utility relative to that reported in adult-onset AAV.

## Patients and methods

### Study cohort and clinical data

Study participants were selected from A Registry of Childhood Vasculitis (ARChiVe), which was established as a pilot time-of-diagnosis repository in 2007^15^ and modified through the Paediatric Vasculitis Initiative (2013) to include follow-up clinical data^7 15^ including demographics, diagnosis, clinical features, laboratory results with organ-specific function tests, medications, disease activity and damage scores, and ANCA status. Eligible participants included children and youth <18 years of age at the time of diagnosis of small vessel primary systemic vasculitis by the treating physician, inclusive of the following AAV subtypes: GPA, MPA, as well as ANCA-positive pauci-immune glomerulonephritis and unclassified primary (small vessel) vasculitis. Individuals with EGPA were excluded from this study as it is exceedingly rare in childhood (representing only 4% of AAV patients in the current registry), and although the ANCA-positive EGPA patients may share some phenotypic features of GPA or MPA patients, the ANCA-negative patients (which are the majority) have a distinct clinical presentation and are usually treated differently. Clinical data for ANCA-negative cases were scrutinised for evidence suggestive of a different diagnosis, resulting in the exclusion of three individuals. In this study, ANCA seropositivity and specificity, as recorded in the registry, were measured by participating sites commonly with an ELISA (392/406 cases) for sera autoantibodies towards MPO and PR3 or via indirect immunofluorescence (14/406 cases) for the presence of cytoplasmic (typically against PR3) or perinuclear (typically against MPO) localised autoantibodies in neutrophils.

### Disease activity, damage and outcomes assessment

Disease activity was quantified in ARChiVe from entered data as previously described^7^ using the paediatric Vasculitis Activity Score (pVAS, range 0–63).^16^ Disease activity in the kidneys was quantified by the renal pVAS (range 0–12). Kidney function was assessed using the estimated glomerular filtration rate (eGFR; mL/min/1.73 m^2^)^17^ and categorised (based on eGFR) according to the Kidney Disease Improving Global Outcomes (KDIGO) guidelines where stage 1 (eGFR≥90) corresponded to normal kidney function, stage 2 (60≤eGFR≤89) indicated mildly reduced kidney function, stage 3 (30≤eGFR≤59) indicated moderately decreased kidney function, stage 4 (15≤eGFR≤29) indicated severely reduced kidney function and stage 5 (eGFR<15) was indicative of kidney failure.^18^

Overall outcome measures as previously described^7^ at 1-year postdiagnosis included achievement of inactive disease (total pVAS≤1), improving disease (≥50% and ≥70% reduction in total pVAS from diagnosis), and the presence of damage (paediatric Vasculitis Damage Index, pVDI≥1) as measured by the pVDI (range 0–76). The pVDI is a clinical tool that records damage in individual organ systems when abnormal features/functions persist for ≥3 months.^19 20^ As damage is considered permanent, scored items are carried forward to subsequent visits. Between 1-year and 2-year postdiagnosis, outcome was also assessed by the occurrence of relapse as reported by the physician or by an increase in the total pVAS from 0 to ≥2, or 1 to ≥4, during this time interval. Relapse was solely defined as a recurrence of disease activity after achievement of inactive disease; a change or escalation of treatment was not required to meet the definition of relapse.

### Statistical analysis

Analyses were performed using R (R Foundation for Statistical Computing, V.4.2.2, Vienna, Austria) and visualised using GraphPad Prism V.9.3.1 for MacOS (GraphPad Software, San Diego, California, USA). Data were presented as median±IQR or mean±SD for continuous variables and counts and percentages for categorical variables.

Linear regression models were fit to investigate the association between ANCA status (the independent factor) and quantitative measures of overall (total pVAS) and kidney-specific (renal pVAS, eGFR) disease as dependent factors. Multivariable logistic regression models were also fit to evaluate associations between ANCA status and categorical measures of overall and organ-specific disease collected as part of the pVAS assessment. For these models, the adjusted ORs, 95% CI and p values were reported. The inverse OR was calculated and reported where appropriate to improve clarity of comparisons. Further, to evaluate longitudinal changes between groups, linear mixed-effects models (with random intercept for patient) for kidney function at diagnosis, 6 months postdiagnosis and 12 months postdiagnosis were fit. These models were stratified by kidney function at diagnosis. Results were summarised as time-specific mean differences between groups (of ANCA positivity and specificity). Finally, using eGFR, participants stratified by ANCA specificity were compared for KDIGO stage at diagnosis using an ordinal logistic regression model. Results from this model were presented as predicted probabilities of outcome level. All models were adjusted for potential confounding variables. These included biological sex assigned at birth, age at diagnosis, and, where appropriate, baseline disease activity and kidney function. Results are interpreted based on the size and direction of effect, and the uncertainty (CI) rather than null hypothesis significance testing.^21^

## Results

### Patients

In our cohort of (n=406) children and adolescents with chronic primary small vessel vasculitis, 58% (n=236) of individuals were PR3-ANCA seropositive, 32% (n=129) of individuals were seropositive for MPO-ANCA and 10% (n=41) were seronegative for ANCA against either antigen. The majority of individuals were Caucasian (53%, 214/406) and female (68%, 278/406). Individuals with a clinical diagnosis (MD diagnosis) of GPA (70%, 282/406) comprised a large proportion of the cohort. Comparatively, 16% (66/406) of individuals were diagnosed with MPA, 5% (22/406) with ANCA-positive pauci-immune glomerulonephritis and 9% (36/406) with unclassified primary vasculitis. Within our cohort, the median age at diagnosis was 14.0 years (IQR: 11.0–16.0).

### ANCA seropositivity is associated with higher overall disease activity at diagnosis

**Figure 1:**
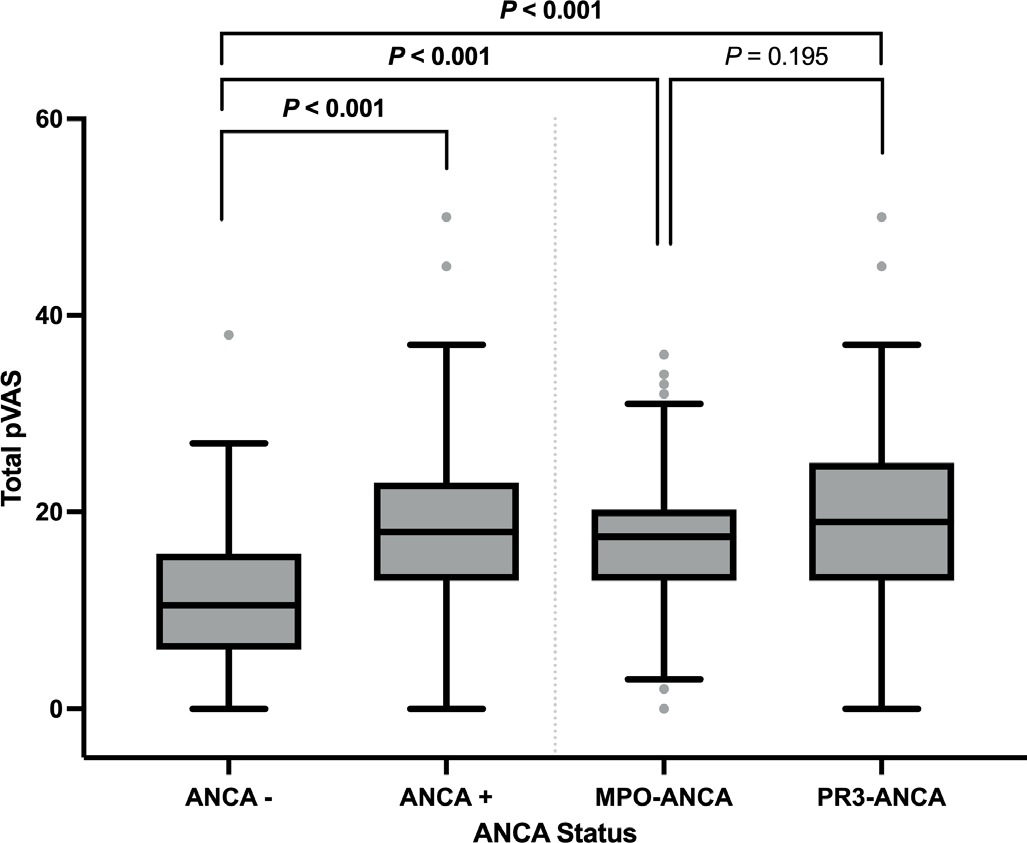
Overall disease activity (pVAS) at diagnosis in paediatric-onset small vessel vasculitis stratified by ANCA status. A linear regression model adjusted for age at the time of diagnosis and sex was used to compare overall disease activity, quantified using the total pVAS (y-axis), at diagnosis in n=398 children and adolescents with small vessel vasculitis (x-axis) stratified according to the absence (ANCA -, n=40) or presence (ANCA +, n=358) of ANCA as well as specificity of ANCA towards MPO (MPO-ANCA, n=126) and PR3 (PR3-ANCA, n=232). Data are presented as boxplots showing the median (solid line) and IQR. See online supplemental table S1 for mean differences. ANCA, anti-neutrophil cytoplasmic antibody; MPO, myeloperoxidase; pVAS, paediatric Vasculitis Activity Score.

Among our cohort, the median disease activity score (total pVAS) at diagnosis from n=398 individuals with available data was 18.0 (IQR: 12.0–22.0). Patients with ANCA seropositivity (vs seronegativity) had a higher average pVAS (mean difference: 6.67, 95% CI 4.17 to 9.16, p<0.001); however, differences between MPO-ANCA and PR3-ANCA were minimal (mean difference: 1.15, 95% CI −0.59 to 2.89, p=0.195) (figure 1 and online supplemental table 1).

10.1136/rmdopen-2024-004315.supp1 Supplementary data

### Disease states within 2 years of diagnosis are not significantly different based on ANCA seropositivity or specificity

Given that ANCA-positive individuals had higher disease activity at diagnosis, we questioned if these individuals may experience sustained disease activity and/or greater accrual of damage within the first 1–2 years of diagnosis. A total of 220 participants (54%) had 1–2 years follow-up data with 68% (150/220) having inactive disease at 1-year postdiagnosis. The likelihood of attaining a state of inactive disease was similar between participants who were seronegative for ANCA, or seropositive for either MPO-ANCA or PR3-ANCA (table 1A). Improvement from diagnosis to 1-year follow-up was observed in a majority of individuals with rates being similar between groups differing in ANCA status (table 1B,C). Rates of permanent damage were higher in individuals seropositive for MPO-ANCA (58%) and PR3-ANCA (60%) compared with ANCA seronegative cases (40%), but adjusted ORs had wide CIs, limiting conclusions (table 1D). Results for relapse occurring between 1 and 2 years after diagnosis were similar regardless of ANCA seropositivity or specificity (table 1E). For the possibility that treatment was associated with disease states, we evaluated if there was an association between ANCA specificity and treatment and found that the adjusted odds of receiving cyclophosphamide, rituximab, azathioprine and methotrexate for remission induction were similar for MPO-ANCA and PR3-ANCA seropositive individuals. For remission maintenance, the odds of receiving cyclophosphamide, rituximab and azathioprine were similar while the odds of receiving methotrexate were slightly higher in the PR3-ANCA group (online supplemental tables 2 and 3).

**Table 1:** Overall outcomes between 1-year and 2-year postdiagnosis in paediatric-onset small vessel vasculitis

ANCA status	% (n)	Adjusted OR (95% CI)	P value	Adjusted OR (95% CI)	P value
(A) Inactive disease at 1 year*					
All participants	68.2 (150/220)				
ANCA-negative	82.4 (14/17)	Reference**	–	2.06 (0.57 to 9.85)	0.67
MPO-ANCA	68.9 (51/74)	0.49 (0.10 to 1.75)	0.31	*Reference*	–
PR3-ANCA	65.9 (85/129)	0.45 (0.10 to 1.60)	0.26	0.93 (0.48 to 1.78)	0.75
(B) Improvement (≥50%) at 1 year†					
All participants	95.8 (206/215)				
ANCA-negative	93.8 (15/16)	*Reference*	–	1.66 (0.17 to 39.73)	0.69
MPO-ANCA	94.4 (68/72)	0.60 (0.03 to 5.81)	0.69	*Reference*	–
PR3-ANCA	96.9 (123/127)	0.58 (0.02 to 5.72)	0.67	0.96 (0.20 to 4.43)	0.95
(C) Improvement (≥70%) at 1 year‡					
All participants	87.0 (187/215)				
ANCA-negative	93.8 (15/16)	*Reference*	–	3.89 (0.61 to 77.24)	0.23
MPO-ANCA	84.7 (61/72)	0.26 (0.01 to 1.64)	0.23	*Reference*	–
PR3-ANCA	87.4 (111/127)	0.24 (0.01 to 1.48)	0.20	0.92 (0.37 to 2.21)	0.85
(D) Damage at 1 year§					
All participants	57.6 (117/203)				
ANCA-negative	40.0 (6/15)	*Reference*	–	0.44 (0.13 to 1.43)	0.18
MPO-ANCA	58.2 (39/67)	2.27 (0.70 to 7.84)	0.18	*Reference*	–
PR3-ANCA	59.5 (72/121)	1.86 (0.59 to 6.16)	0.29	0.82 (0.42 to 1.58)	0.55
(E) Relapse between 1 and 2 years¶					
All participants	10.7 (16/150)				
ANCA-negative	7.1 (1/14)	*Reference*	–	0.71 (0.03 to 5.22)	0.77
MPO-ANCA	11.8 (6/51)	1.41 (0.19 to 29.03)	0.77	*Reference*	–
PR3-ANCA	10.6 (9/85)	1.64 (0.23 to 33.44)	0.67	1.16 (0.36 to 3.96)	0.80

*Inactive disease was defined as a total pVAS≤1 at 1-year postdiagnosis.

†Improvement was defined as a ≥50% reduction in total pVAS from diagnosis to 1-year postdiagnosis.

‡Improvement was defined as a ≥70% reduction in total pVAS from diagnosis to 1-year postdiagnosis.

§Damage was defined as a pVDI ≥1 at 1-year postdiagnosis.

¶Relapsing disease was defined as an increase in total pVAS from 0 to ≥2 or 1 to ≥4 from 1 to 2 years postdiagnosis, alongside relapses noted by the treating physician during this time interval.

**Reference indicates the reference group to which the ORs are being compared.

ANCA, anti-neutrophil cytoplasmic antibody; MPO, myeloperoxidase; pVAS, paediatric Vasculitis Activity Score; pVDI, paediatric Vasculitis Damage Index.

### MPO-ANCA seropositivity is associated with a higher prevalence of kidney involvement and a lower prevalence of ENT involvement than PR3-ANCA seropositivity

**Figure 2:**
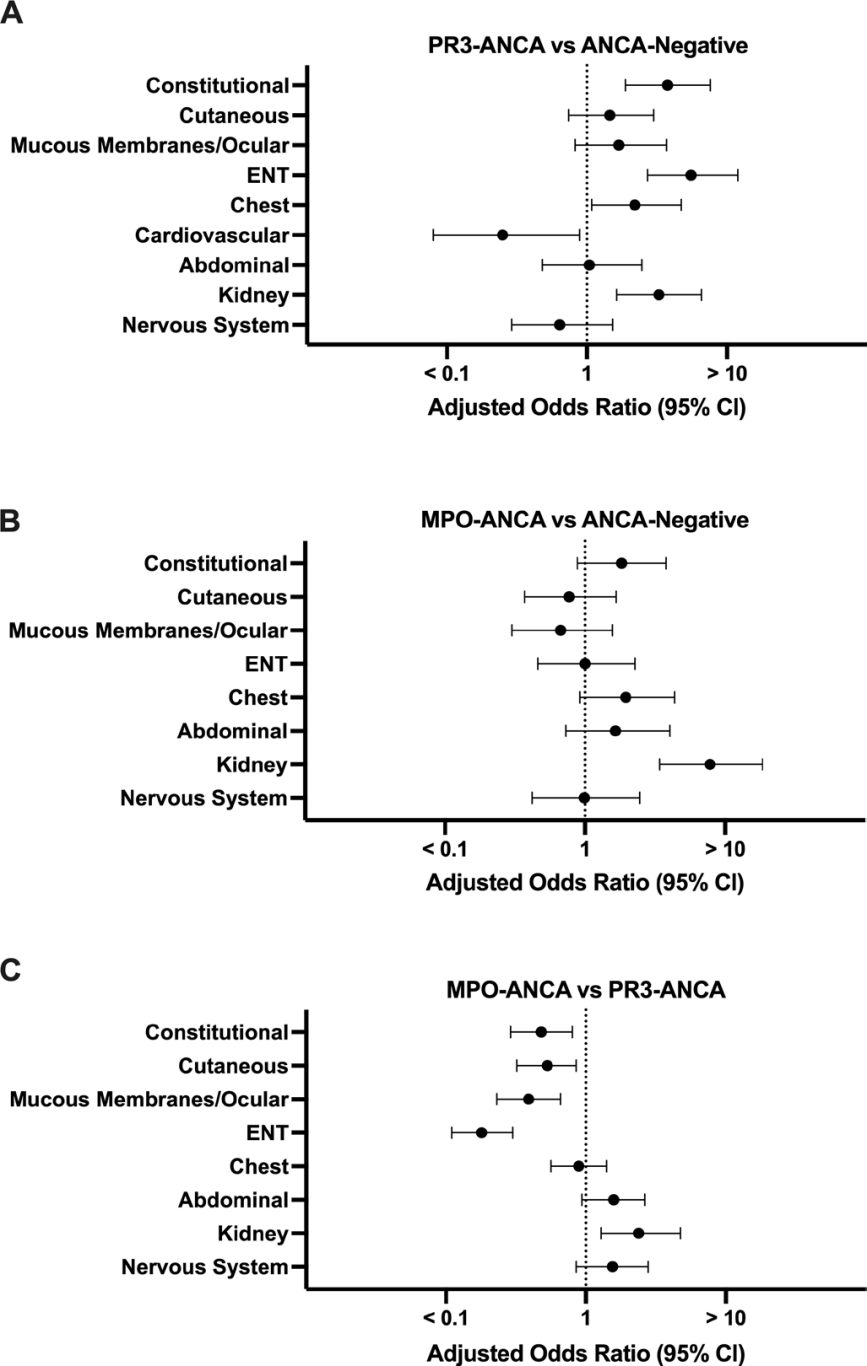
Time of diagnosis organ involvement associated with ANCA positivity and specificity. A logistic regression model adjusted for age at the time of diagnosis and sex was used to evaluate differences in organ involvement (y-axis; organ system) at diagnosis between children stratified by ANCA status (ANCA negative, n=40; MPO-ANCA, n=126; PR3-ANCA, n=232). (A) PR3-ANCA seropositivity and (B) MPO-ANCA seropositivity are compared with ANCA seronegative cases. (C) MPO-ANCA seropositive and PR3-ANCA seropositive cases are compared. Data are presented as forest plots with the OR (x-axis, logarithmic scale) and 95% CI. ANCA, anti-neutrophil cytoplasmic antibody; ENT, ear, nose and throat; MPO, myeloperoxidase.

Although overall disease activity and outcomes were comparable between participants seropositive for MPO- and PR3-ANCA, we sought to assess potential phenotypic differences in organ involvement related to ANCA antigen positivity and specificity (figure 2 and online supplemental table 4).

Kidney involvement was noted in a majority of participants with MPO-ANCA (88%, 111/126) and PR3-ANCA (77%, 179/232), as well as half of the individuals with ANCA-negative vasculitis (50%, 20/40) (online supplemental table 4). Compared with ANCA-negative vasculitis cases, the adjusted odds of participants having kidney involvement were 7.8 (95% CI 3.4 to 18.5, p<0.001) and 3.3 (95% CI 1.6 to 6.6, p<0.001) times higher in individuals with MPO-ANCA and PR3-ANCA, respectively. Additionally, the adjusted odds of kidney involvement were 2.4 (95% CI 1.3 to 4.7, p=0.008) times higher in participants who were seropositive for MPO-ANCA versus PR3-ANCA, which was attributable to certain clinical manifestations, specifically hypertension, proteinuria and haematuria/the presence of RBC casts.

Conversely, rates of ear, nose and throat (ENT) involvement were highest in PR3-ANCA-seropositive participants, characterised by significantly higher adjusted odds of bloody nasal discharge/crusts/ulcers/granuloma, paranasal sinus involvement and conductive hearing loss (online supplemental table 4). PR3-ANCA positive individuals also had 2.6 (95% CI 1.5 to 4.3, p<0.001) times the adjusted odds of mucous membranes/ocular involvement, which was predominantly characterised by the presence of mouth ulcers/granulomata, compared with MPO-ANCA-positive cases.

### MPO-ANCA seropositivity is more often associated with kidney insufficiency at diagnosis compared with PR3-ANCA seropositivity

To evaluate associations between ANCA status and the extent of kidney disease at diagnosis, we compared participants stratified by ANCA status for both kidney disease activity measured by the renal subcomponent of pVAS, and kidney function measured by eGFR.

**Figure 3:**
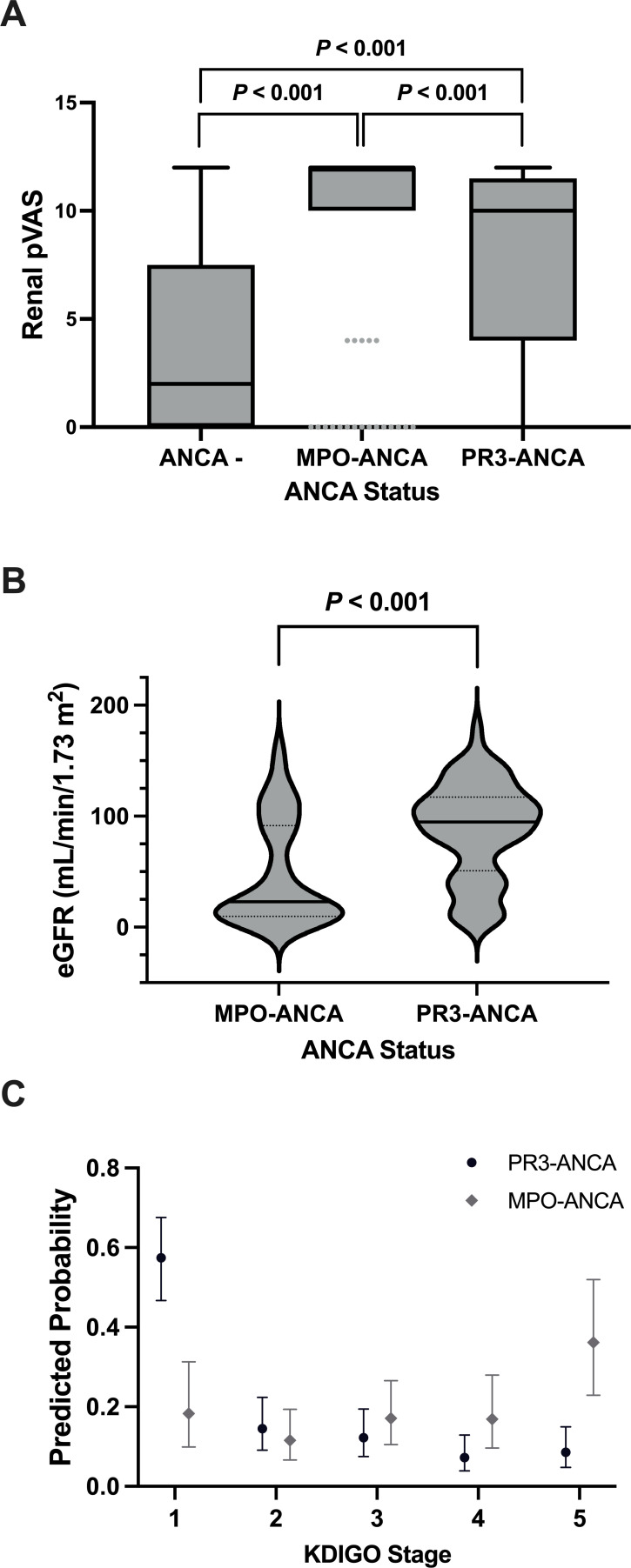
Kidney disease at diagnosis in paediatric-onset small vessel vasculitis stratified by ANCA status. A linear regression model was used to compare (A) kidney-specific disease activity, as measured by the renal pVAS (y-axis; median) (x-axis: ANCA-negative, n=43; MPO-ANCA, n=126; PR3-ANCA, n=232) and (B) kidney function, as measured by the eGFR (y-axis; median) (x-axis: MPO-ANCA, n=39; PR3-ANCA, n=84), at the time of diagnosis in children and adolescents with small vessel vasculitis. (C) An ordinal logistic regression model was used to compare the predicted probability (y-axis) of having (x-axis) normal kidney function (KDIGO stage 1, eGFR≥90), mildly reduced kidney function (KDIGO stage 2, 60≤eGFR≤89), moderately reduced kidney function (KDIGO stage 3, 30≤eGFR≤59), severely reduced kidney function (KDIGO stage 4, 15≤eGFR≤29) and kidney failure (KDIGO stage 5, eGFR<15) at diagnosis between MPO-ANCA (grey, n=39) and PR3-ANCA (black, n=84) seropositive AAV. All models were adjusted for age at diagnosis and sex as baseline variables. Data are presented as (A) boxplots with the median±IQR, (B) violin plots with the median (solid line) and IQR and (C) predicted probability±95% CI. AAV, ANCA-associated vasculitis; ANCA, anti-neutrophil cytoplasmic antibody; eGFR, estimated glomerular filtration rate; KDIGO, Kidney Disease Improving Global Outcomes; MPO, myeloperoxidase; pVAS, paediatric Vasculitis Activity Score.

The median renal pVAS of participants with MPO-ANCA was 12.0 (IQR: 10.0–12.0) compared with 10.0 (4.0–10.5) in individuals with PR3-ANCA and 2.0 (0.0–6.5) for participants with ANCA-negative vasculitis. The average renal pVAS of participants seropositive for either MPO-ANCA (MPO-ANCA vs ANCA-negative, mean±SD: 9.6±3.9 vs 3.9±4.5, mean difference: 5.71, 95% CI 4.13 to 7.29, p<0.001) or PR3-ANCA (PR3-ANCA vs ANCA-negative, mean±SD: 7.5±4.6 vs 3.9±4.5, mean difference: 3.45, 95% CI 1.98 to 4.93, p<0.001) was higher than ANCA-seronegative participants (figure 3A). Further, the average renal pVAS of MPO-ANCA-positive cases was higher than that of participants with PR3-ANCA (MPO-ANCA vs PR3-ANCA, mean±SD: 9.6±3.9 vs 7.5±4.6, mean difference: 2.26, 95% CI 1.26 to 3.26, p<0.001) (figure 3A),

Kidney function was assessed in 123 participants (MPO-ANCA, n=39; PR3-ANCA, n=84) with eGFR data at diagnosis. MPO-ANCA seropositive individuals had a lower mean eGFR compared with PR3-ANCA seropositive cases (MPO-ANCA vs PR3-ANCA, mean±SD: 48.9±47.8 vs 87.4±43.6, mean difference: −42.70, 95% CI −60.15 to −25.26, p<0.001) (figure 3B). In accordance with significantly lower eGFR, the adjusted odds of participants having a higher KDIGO stage (ie, worse kidney function) at diagnosis were 6.04 (95% CI 2.77 to 13.57, p<0.001) times higher in participants who were seropositive for MPO-ANCA versus PR3-ANCA. MPO-ANCA seropositive participants had a higher probability of being in kidney failure (KDIGO stage 5) at diagnosis than PR3-ANCA seropositive participants (0.36 (95% CI 0.23 to 0.52) vs 0.09 (95% CI 0.05 to 0.15)). Conversely, the probability of having normal kidney function (KDIGO stage 1) was higher in PR3-ANCA seropositive participants than in participants positive for MPO-ANCA (0.57 (95% CI 0.47 to 0.68) vs 0.18 (95% CI 0.10 to 0.31)) (figure 3C).

### MPO-ANCA seropositivity is associated with a more favourable kidney disease trajectory compared with PR3-ANCA seropositivity in patients with severely reduced kidney function or kidney failure at diagnosis

To determine if kidney disease trajectories differ between MPO-ANCA and PR3-ANCA-seropositive participants matched for kidney function at diagnosis, we evaluated the change in eGFR from diagnosis to follow-up at 6 and 12 months in participants with normal to mildly reduced kidney function (KDIGO stages 1–2), moderately reduced kidney function (KDIGO stage 3) and severely reduced kidney function to kidney failure (KDIGO stages 4–5) at diagnosis. There were 74, 16 and 33 patients with KDIGO stages 1–2, 3 and 4–5, respectively.

**Figure 4:**
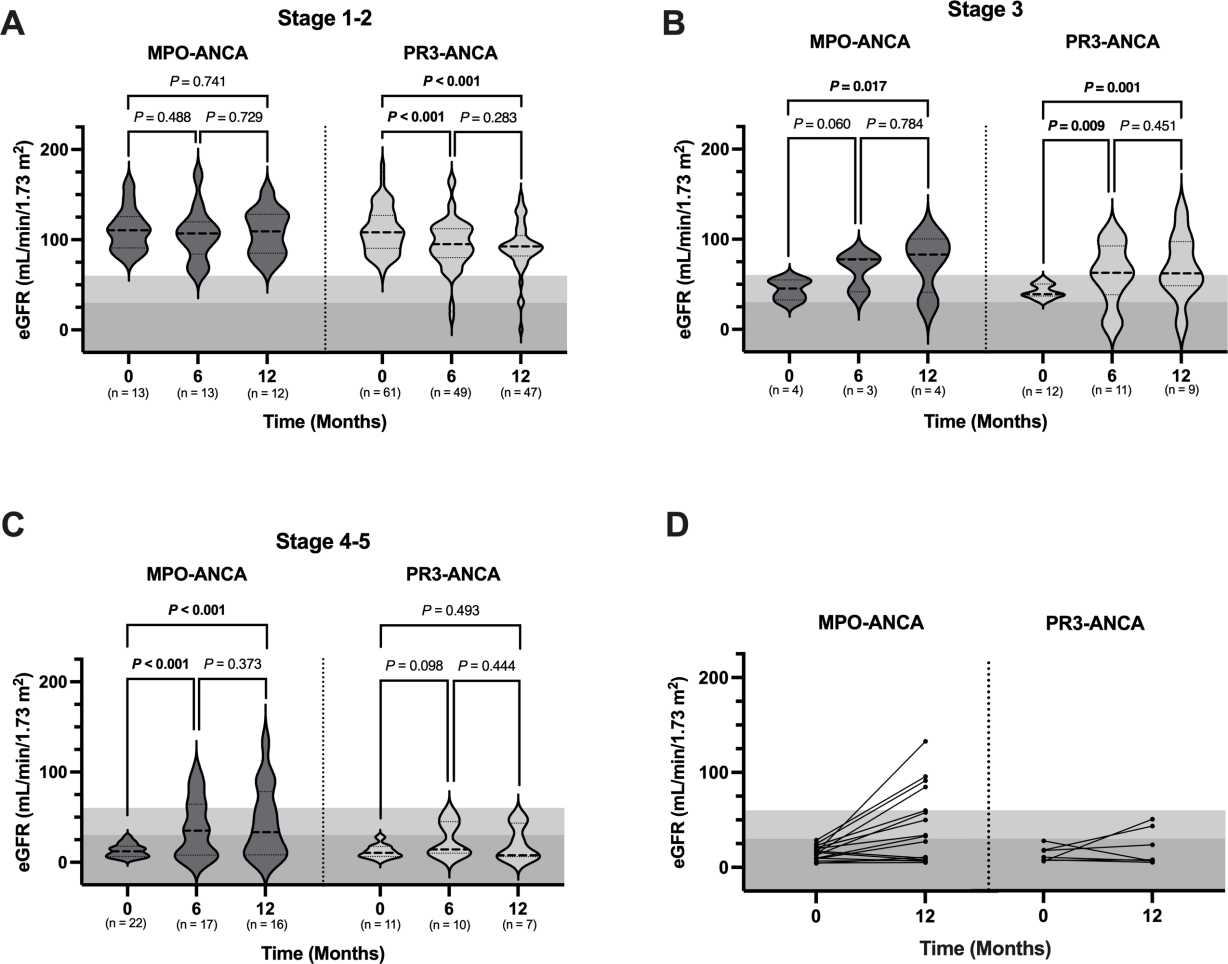
Kidney disease trajectory from diagnosis to 1-year postdiagnosis in paediatric-onset AAV stratified by KDIGO stage at diagnosis and ANCA specificity. A linear mixed-effects model, adjusted for eGFR and age at diagnosis, alongside sex, was used to compare changes in kidney function, quantified using eGFR (y-axis) at the time of diagnosis, 6 months postdiagnosis and 12 months postdiagnosis (x-axis) between MPO-ANCA and PR3-ANCA seropositive paediatric patients with (A) normal to mildly reduced kidney function (KDIGO stages 1–2, eGFR≥60), (B) moderately reduced kidney function (KDIGO stage 3, 30≤eGFR≤59) or (C) severely reduced kidney function or kidney failure (KDIGO stages 4–5, eGFR≤29) at the time of diagnosis. (D) eGFR at diagnosis and 1-year postdiagnosis are also shown for a subset of participants with data availability who had severely reduced kidney function or kidney failure at diagnosis. Graphs are shaded to visualise KDIGO stages 1–2 (white), 3 (grey) and 4–5 (dark grey). Numbers in brackets on the x-axis (A–C) are individual patients. Data are presented as violin plots with the median (dotted line) and IQR. AAV, ANCA-associated vasculitis; ANCA, anti-neutrophil cytoplasmic antibody; eGFR, estimated glomerular filtration rate; KDIGO, Kidney Disease Improving Global Outcomes; MPO, myeloperoxidase.

In children and adolescents with normal to mildly reduced kidney function (KDIGO stages 1–2) at the time of diagnosis, there was a decline (worsening) in mean eGFR in PR3-ANCA-positive individuals at 6 and 12 months postdiagnosis (6 months vs diagnosis, mean±SD: 95.6±27.6 vs 109.9±25.3, mean difference: −14.97, 95% CI −22.10 to −7.82, p<0.001; 12 months vs diagnosis, mean±SD=91.7 ± 27.6 vs 109.9±25.3, mean difference: −19.20, 95% CI: −26.40 to −11.90, p<0.001), but no such pattern for MPO-ANCA seropositive cases (figure 4A and online supplemental table 5). In those with indications of moderately reduced kidney function (KDIGO stage 3), both PR3-ANCA and MPO-ANCA seropositivity exhibited an increase (improvement) in eGFR at 12 months (PR3-ANCA: 12 months vs diagnosis, 68.7±35.9 vs 42.1±7.6, mean difference: 26.20, 95% CI 10.18 to 42.20, p=0.001) (MPO-ANCA: 12 months vs diagnosis, 74.8±32.3 vs 44.3±11.7, mean difference: 30.50, 95% CI 5.47 to 55.60, p=0.017) (figure 4B and online supplemental table 5). Finally, for participants experiencing severely reduced kidney function or kidney failure (KDIGO stages 4–5) at diagnosis, we observed an increase in the eGFR of MPO-ANCA seropositive individuals from the time of diagnosis to 6 and 12 months postdiagnosis (6 months vs diagnosis, 38.2±32.4 vs 13.0±7.1, mean difference: 23.50, 95% CI 12.57 to 34.40, p<0.001; 12 months vs diagnosis, 44.6±39.4 vs 13.0±7.1, mean difference: 28.76, 95% CI 17.60 to 39.90, p<0.001) (figure 4C and online supplemental table 5). The PR3-ANCA seropositive group also showed an increase, but results were less certain with CIs containing moderate decreases.

Based on the average eGFR in MPO-ANCA seropositive individuals at each time point, an accompanying shift to KDIGO stage 3 (moderately reduced kidney function) was observed. In contrast, there were no significant differences between eGFR at diagnosis, 6 months postdiagnosis, and 12 months postdiagnosis in PR3-ANCA seropositive participants who have severely reduced kidney function or kidney failure (KDIGO stages 4–5) at diagnosis.

In a subgroup analysis of 23 patients (16 MPO-ANCA positive and 7 PR3-ANCA positive) with severely reduced kidney function or kidney failure at diagnosis and available paired data (eGFR) at 12 months postdiagnosis, we found that 56% (9/16) of MPO-ANCA seropositive participants showed improvement in kidney function from KDIGO stages 4–5 at diagnosis to KDIGO stage 3 for 31% of individuals and stages 1–2 for an additional 25% by 1 year (figure 4D). In contrast, only 28% (2/7) of PR3-ANCA seropositive individuals with severely reduced kidney function or kidney failure (KDIGO stages 4–5) at diagnosis achieved KDIGO stage 3 by 12 months postdiagnosis and no PR3-ANCA seropositive individual improved to KDIGO stages 1–2.

## Discussion

Untreated AAV can be organ-threatening and life-threatening and, although modern treatments (namely glucocorticoids in combination with either cyclophosphamide or rituximab) have dramatically lowered mortality rates, the prolonged use of these immunosuppressive agents in high doses is associated with both short-term and long-term toxicity risks.^3 22^ As a result, AAV remains a chronic, remitting and relapsing disease, with continuous accumulation of damage from treatment toxicity and the disease itself. In adult-onset AAV, ANCA positivity and specificity facilitate treatment decision-making by predicting disease prognosis. In this study, we evaluated associations between ANCA status, disease activity at diagnosis and 1–2 years outcomes in 406 children and adolescents with small vessel vasculitis inclusive of AAV.

In our cohort, we observed similar disease activity at diagnosis in children seropositive for MPO-ANCA and PR3-ANCA that was significantly higher than disease activity at the onset of ANCA-negative vasculitis. These findings are consistent with a previous study in a cohort of 84 adults with pauci-immune glomerulonephritis that demonstrated elevated disease activity in ANCA-positive versus ANCA-negative individuals.^23^ Similarly, in 105 paediatric AAV patients,^7^ improving disease and achievement of disease inactivity occurred, although not without damage, for the majority of individuals within 1 year of diagnosis. Further, we demonstrate that ANCA positivity and specificity do not have differential effects on these outcomes (ie, improvement, achievement of inactive disease and damage). Similarly, and in contrast to a higher risk of relapsing disease in adult-onset PR3-ANCA-positive AAV,^8^ neither ANCA positivity nor specificity was predictive of relapse in paediatric AAV.

Although disease activity at diagnosis was similar in individuals in our cohort with either PR3-ANCA or MPO-ANCA, we noted differences in clinical manifestations that are consistent with observations in adult-onset AAV.^24^ Namely, multiorgan involvement comprising both renal and extrarenal organ systems is seen more frequently in children and adolescents with PR3-ANCA while individuals with MPO-ANCA typically present with kidney-limited disease. In comparing the current study to previous reports in adult-onset AAV, specifically the RAVE trial describing baseline clinical manifestations of adult-onset AAV, the mucous membranes/ocular and ENT systems are more frequently involved in PR3-ANCA versus MPO-ANCA seropositive disease, in both paediatric-onset and adult-onset cases.^12^ Focusing on the frequency of pulmonary involvement, no differences were observed between PR3-ANCA and MPO-ANCA seropositive adults in the RAVE trial cohort^12^ or in our paediatric small vessel vasculitis cohort. In contrast, cutaneous involvement, shown in this study to be more frequent in paediatric PR3-ANCA seropositive cases, does not exhibit similar differences in adults stratified by ANCA specificity.^12 24^

Although historically, the prevalence of kidney involvement did not differ in adults stratified by ANCA specificity, more recent studies describe significant differences^12 25 26^ that are aligned with our findings in this paediatric AAV cohort of more frequent kidney involvement at diagnosis in individuals with MPO-ANCA relative to PR3-ANCA seropositivity. Both MPO-ANCA and PR3-ANCA seropositive cases also exhibited higher frequencies of kidney involvement than ANCA-negative vasculitis. Thus, unlike other organ systems, the frequency of kidney disease among paediatric small vessel vasculitis patients varied depending on ANCA positivity and specificity; specifically, kidney involvement was increasingly more frequent from ANCA-negative, to PR3-ANCA-positive, to MPO-ANCA-positive cases. In our cohort, we also observed worse kidney function at diagnosis (as indicated by lower eGFR and a higher probability of meeting criteria for KDIGO stage 5, kidney failure) in individuals with MPO-ANCA, with the opposite observed in PR3-ANCA seropositive patients. This is consistent with findings in adult studies.^27^

It is noteworthy that while patients with MPO-ANCA positivity were more likely to have worse kidney disease at outset compared with PR3-ANCA positive patients, they were also more likely to have more pronounced improvements in kidney function; these improvements were more pronounced for individuals presenting with severely reduced kidney function or kidney failure (KDIGO stages 4–5) compared with PR3-ANCA seropositive individuals with similarly poor baseline kidney function. The corollary to this is that PR3-ANCA positive kidney disease may be more refractory in nature compared with MPO-ANCA positive kidney disease of similar severity as evident by less substantive improvements (with respect to patient numbers and absolute change in eGFR) in kidney function between diagnosis and 12 months irrespective of baseline KDIGO stage.

Our findings, while compelling, are not without potential limitations. The scoring of overall and organ-specific disease activity via the pVAS is susceptible to errors despite availability of a comprehensive manual and instructional video to support accurate completion of the pVAS assessment form. Despite having the largest reported paediatric AAV study cohort, the relatively small number of ANCA-negative cases in our cohort, combined with missing data for some variables of interest, may have compromised statistical power (eg, missing eGFR data precluded comparisons with the ANCA-negative subgroup). Given that the majority of the cohort was Caucasian, language and cultural barriers may have hindered the recruitment, enrolment and retention of racial and ethnic minorities. As a result, generalisability of these data to non-white populations may be limited.

In summary, findings from this study affirm, for the first time in paediatric-onset AAV, associations described in adult-onset AAV between MPO-ANCA seropositivity and a higher prevalence of severe, kidney-limited disease compared with individuals with PR3-ANCA seropositivity. While ANCA positivity and specificity were not predictive of overall improvement in disease within 1 year of diagnosis, and in spite of more severe disease at onset, individuals with MPO-ANCA positive kidney disease showed substantially greater improvement over 1 year compared with PR3-ANCA positive cases. Finally, our data do not recapitulate findings in adult-onset AAV that PR3-ANCA seropositivity is predictive of relapsing disease.

## Data Availability

Data are available on reasonable request. The data that support the findings of this study are available from the corresponding author on reasonable request.
