# Supplementary material for: Predictive utility of ANCA positivity and antigen specificity in the assessment of kidney disease in paediatric-onset small vessel vasculitis
Source: RMD Open. 2024 Jun 17;10(2):e004315. doi: 10.1136/rmdopen-2024-004315 (PMC11184192; doi:10.1136/rmdopen-2024-004315)
Supplement: Supplementary data [file rmdopen-2024-004315supp001.pdf]

SUPPLEMENTAL MATERIAL

Supplementary Table 1. Differences in overall disease activity (total pVAS) at diagnosis between pediatric-onset small vessel vasculitis stratified by ANCA status.

	**Mean Total pVAS ± SD**	**Mean Difference (95% CI)**	***P*-value**
ANCA-Positive *vs.* ANCA-Negative	18.3 ± 7.7 *vs.* 11.7 ± 7.5	6.67 (4.17, 9.16)	<0.001
MPO-ANCA *vs.* ANCA-Negative	17.2 ± 6.4 *vs.* 11.7 ± 7.5	5.91 (3.17, 8.65)	<0.001
PR3-ANCA *vs.* ANCA-Negative	19.0 ± 8.2 *vs.* 11.7 ± 7.5	7.06 (4.50, 9.62)	<0.001
PR3-ANCA *vs.* MPO-ANCA	19.0 ± 8.2 *vs.* 17.2 ± 6.4	1.15 (-0.59, 2.89)	0.195

Supplementary Table 2. Treatment for remission-induction in pediatric-onset small vessel vasculitis stratified by ANCA status.

	**ANCA Status**				**PR3-ANCA vs. MPO-ANCA**	
	**All** (n = 384)	**ANCA-negative** (n = 40)	**MPO-ANCA** (n = 121)	**PR3-ANCA** (n = 223)	**Adjusted OR (95% CI)**	***P*-value**
Cyclophosphamide	243 (63.3)	12 (30.0)	78 (64.5)	153 (68.6)	1.20 (0.75, 1.92)	0.434
Rituximab	78 (20.3)	2 (5.0)	30 (24.8)	46 (20.6)	0.79 (0.47, 1.34)	0.374
Azathioprine	11 (2.9)	1 (2.5)	3 (2.5)	7 (3.1)	1.27 (0.35, 6.00)	0.729
Methotrexate	51 (13.3)	8 (20.0)	11 (9.1)	32 (14.3)	1.68 (0.84, 3.60)	0.162

Data are presented as the absolute number (%) of individuals in a given category.
Abbreviations: OR: odds ratio, CI: confidence interval

Supplementary Table 3. Treatment for remission-maintenance (1-year) in pediatric-onset small vessel vasculitis stratified by ANCA status.

	**ANCA Status**				**PR3-ANCA vs. MPO-ANCA**	
	**All** (n = 223)	**ANCA-negative** (n = 15)	**MPO-ANCA** (n = 79)	**PR3-ANCA** (n = 129)	**Adjusted OR (95% CI)**	***P*-value**
Cyclophosphamide	51 (22.9)	3 (20.0)	21 (26.6)	27 (20.9)	0.73 (0.38, 1.42)	0.349
Rituximab	54 (24.2)	3 (20.0)	24 (30.4)	27 (20.9)	0.61 (0.32, 1.15)	0.126
Azathioprine	87 (39.0)	4 (26.7)	26 (32.9)	57 (44.2)	1.61 (0.91, 2.92)	0.108
Methotrexate	42 (18.8)	5 (33.3)	8 (10.1)	29 (22.5)	**2.57 (1.16, 6.34)**	**0.027**

Data are presented as the absolute number (%) of individuals in a given category.
Abbreviations: OR: odds ratio, CI: confidence interval

Supplementary Table 4. Organ involvement and clinical characteristics associated with ANCA type in pediatric-onset small vessel vasculitis at diagnosis.

				Logistic Regression Model					
	% (n)			PR3-ANCA vs ANCA-Negative		MPO-ANCA vs ANCA-Negative		MPO-ANCA vs PR3-ANCA	
	ANCA-Negative (n = 40)	MPO-ANCA (n = 126)	PR3-ANCA (n = 232)	Adjusted OR (95% CI)	*P*-value	Adjusted OR (95% CI)	*P*-value	Adjusted OR (95% CI)	*P*-value
Constitutional involvement	47.5 (19)	62.7 (79)	76.7 (178)	**3.77 (1.88, 7.62)**	**<0.001**	1.82 (0.88, 3.79)	0.109	**0.48 (0.29, 0.80)**	**0.004**
Myalgia	12.5 (5)	15.9 (20)	16.8 (39)	1.41 (0.56, 4.33)	0.475	1.47 (0.54, 4.73)	0.475	1.04 (0.55, 1.92)	0.893
Arthralgia or arthritis	42.5 (17)	38.1 (48)	60.8 (141)	**2.22 (1.12, 4.47)**	**0.023**	0.88 (0.42, 1.84)	0.722	**0.39 (0.24, 0.63)**	**<0.001**
Fever ≥ 38°C	27.5 (11)	23.0 (29)	32.8 (76)	1.28 (0.62, 2.82)	0.522	0.68 (0.30, 1.60)	0.366	**0.53 (0.31, 0.90)**	**0.021**
Weight loss ≥ 5% of body weight	15.0 (6)	29.4 (37)	43.1 (100)	**4.19 (1.80, 11.48)**	**0.002**	**2.70 (1.09, 7.68)**	**0.043**	**0.64 (0.39, 1.04)**	**0.075**

Cutaneous involvement	37.5 (15)	31.0 (39)	46.1 (107)	1.46 (0.74, 2.99)	0.281	0.77 (0.37, 1.67)	0.506	**0.53 (0.32, 0.85)**	**0.009**
Polymorphous exanthema	2.5 (1)	4.8 (6)	9.5 (22)	4.10 (0.82, 74.62)	0.174	1.82 (0.29, 35.24)	0.587	0.44 (0.15, 1.12)	0.104
Livedo	0.0 (0)	3.2 (4)	2.6 (6)	-	-	-	-	0.89 (0.21, 3.31)	0.858
Panniculitis	7.5 (3)	3.2 (4)	0.4 (1)	**0.06 (2.80e-03, 0.47)**	**0.015**	0.46 (0.09, 2.50)	0.337	8.00 (1.12, 160.57)	0.069
Purpura	22.5 (9)	13.5 (17)	32.8 (76)	1.69 (0.79, 3.95)	0.194	0.54 (0.22, 1.40)	0.189	**0.32 (0.17, 0.58)**	**<0.001**
Skin nodules	7.5 (3)	4.0 (5)	8.6 (20)	1.16 (0.37, 5.16)	0.816	0.62 (0.14, 3.15)	0.524	0.53 (0.17, 1.39)	0.228
Infarct (nail edge lesion, splinter hemorrhage)	2.5 (1)	1.6 (2)	5.2 (12)	2.14 (0.39, 39.77)	0.476	0.80 (0.07, 17.73)	0.861	0.38 (0.06, 1.46)	0.214
Ulcer (full-thickness necrosis)	0.0 (0)	0.8 (1)	3.4 (8)	-	-	-	-	0.21 (0.01, 1.21)	0.147
Gangrene (extensive necrosis)	0.0 (0)	0.0 (0)	1.3 (3)	-	-	-	-	-	-
Other skin vasculitis	0.0 (0)	0.0 (0)	0.0 (0)	-	-	-	-	-	-
Mucous membranes/ocular involvement	27.5 (11)	20.6 (26)	39.2 (91)	1.69 (0.82, 3.71)	0.165	0.67 (0.30, 1.57)	0.337	**0.39 (0.23, 0.66)**	**<0.001**
Mouth ulcers/granulomata	0.0 (0)	4.0 (5)	18.5 (43)	**-**	**-**	-	-	**0.18 (0.06, 0.45)**	**<0.001**
Genital ulcers	0.0 (0)	0.8 (1)	0.4 (1)	-	-	-	-	1.51 (0.06, 40.10)	0.777
Adnexal inflammation	0.0 (0)	0.0 (0)	0.0 (0)	-	-	-	-	-	-
Significant proptosis	0.0 (0)	0.0 (0)	0.0 (0)	-	-	-	-	-	-
Red eye (epi)scleritis	5.0 (2)	3.2 (4)	10.8 (25)	2.02 (0.56, 12.97)	0.356	0.75 (0.14, 5.60)	0.745	0.37 (0.10, 1.02)	0.079
Red eye conjunctivitis/blepharitis/keratitis	7.5 (3)	6.3 (8)	12.5 (29)	1.61 (0.53, 7.02)	0.453	0.83 (0.22, 4.02)	0.800	0.52 (0.21, 1.18)	0.134
Uveitis	5.0 (2)	0.0 (0)	3.9 (9)	0.67 (0.16, 4.57)	0.623	-	-	-	-
Blurred vision	15.0 (6)	4.8 (6)	3.0 (7)	**0.18 (0.06, 0.61)**	**0.004**	0.30 (0.09, 1.05)	0.056	1.65 (0.50, 5.29)	0.394
Sudden visual loss	0.0 (0)	0.0 (0)	0.0 (0)	-	-	-	-	-	-
Retinal vasculitis/retinal vessel thrombosis/retinal exudates/hemorrhages	0.0 (0)	0.0 (0)	0.4 (1)	-	-	-	-	-	-
ENT involvement	30.0 (12)	27.8 (35)	70.3 (163)	**5.55 (2.71, 12.01)**	**<0.001**	1.00 (0.46, 2.28)	0.991	**0.18 (0.11, 0.30)**	**<0.001**
Bloody nasal discharge/crusts/ulcers/granuloma	12.5 (5)	15.9 (20)	54.3 (126)	**8.29 (3.40, 24.88)**	**<0.001**	1.45 (0.54, 4.65)	0.490	**0.18 (0.10, 0.30)**	**<0.001**
Paranasal sinus involvement	10.0 (4)	15.1 (19)	37.9 (88)	**5.88 (2.24, 20.28)**	**0.001**	1.80 (0.62, 6.56)	0.319	**0.31 (0.17, 0.54)**	**<0.001**
Subglottic stenosis/hoarseness/stridor	12.5 (5)	4.8 (6)	8.6 (20)	0.69 (0.25, 2.19)	0.483	0.41 (0.11, 1.52)	0.165	0.60 (0.21, 1.49)	0.298
Conductive hearing loss	0.0 (0)	1.6 (2)	12.1 (28)	-	-	-	-	**0.13 (0.02, 0.46)**	**0.007**
Sensorineural hearing loss	2.5 (1)	1.6 (2)	3.0 (7)	1.13 (0.19, 21.65)	0.912	0.82 (0.08, 17.97)	0.870	0.72 (0.10, 3.19)	0.695
Chest involvement	30.0 (12)	43.7 (55)	49.6 (115)	**2.20 (1.08, 4.70)**	**0.034**	1.95 (0.92, 4.36)	0.090	0.89 (0.56, 1.41)	0.616
Wheeze or expiratory dyspnea	7.5 (3)	7.9 (10)	7.8 (18)	0.97 (0.31, 4.33)	0.968	1.18 (0.33, 5.53)	0.811	1.21 (0.50, 2.79)	0.658
Endobronchial/endotracheal involvement	5.0 (2)	0.8 (1)	3.0 (7)	0.65 (0.15, 4.48)	0.596	0.17 (0.01, 1.81)	0.150	0.26 (0.01, 1.53)	0.212
Nodules or cavities	0.0 (0)	0.0 (0)	0.0 (0)	-	-	-	-	-	-
Pleural effusion/pleurisy	7.5 (3)	10.3 (13)	9.9 (23)	1.35 (0.44, 5.93)	0.641	1.67 (0.50, 7.67)	0.445	1.24 (0.57, 2.60)	0.574
Infiltrate	0.0 (0)	0.0 (0)	0.0 (0)	-	-	-	-	-	-
Massive hemoptysis/alveolar hemorrhage	15.0 (6)	28.6 (36)	34.9 (81)	**2.83 (1.21, 7.77)**	**0.026**	2.39 (0.97, 6.84)	0.076	0.85 (0.51, 1.39)	0.513
Respiratory failure	12.5 (5)	5.6 (7)	12.1 (28)	0.88 (0.34, 2.73)	0.799	0.46 (0.14, 1.66)	0.215	0.53 (0.20, 1.23)	0.159
Cardiovascular involvement	12.5 (5)	0.8 (1)	3.4 (8)	**0.25 (0.08, 0.89)**	**0.024**	**0.04 (1.90e-03, 0.27)**	**0.005**	0.15 (0.01, 0.93)	0.088

Loss of pulses	2.5 (1)	0.0 (0)	0.9 (2)	0.29 (0.03, 6.44)	0.323	-	-	-	-
Bruits over accessible arteries	0.0 (0)	0.0 (0)	0.9 (2)	-	-	-	-	-	-
Blood pressure discrepancy	0.0 (0)	0.0 (0)	0.0 (0)	-	-	-	-	-	-
Caudication of extremities	5.0 (2)	0.0 (0)	0.9 (2)	0.17 (0.02, 1.50)	0.087	-	-	-	-
Ischaemic cardiac pain	0.0 (0)	0.0 (0)	0.0 (0)	-	-	-	-	-	-
Cardiomyopathy	5.0 (2)	0.0 (0)	0.0 (0)	-	-	-	-	-	-
Congestive cardiac failure	2.5 (1)	0.0 (0)	0.0 (0)	-	-	-	-	-	-
Valvular heart disease	0.0 (0)	0.0 (0)	0.0 (0)	-	-	-	-	-	-
Pericarditis	5.0 (2)	0.8 (1)	1.3 (3)	0.27 (0.04, 2.13)	0.164	0.12 (0.01, 1.37)	0.098	0.44 (0.02, 3.82)	0.496
Abdominal involvement	22.5 (9)	31.7 (40)	25.0 (58)	1.04 (0.48, 2.47)	0.919	1.65 (0.73, 4.03)	0.250	1.58 (0.94, 2.64)	0.083
Abdominal pain	15.0 (6)	29.4 (37)	22.0 (51)	1.48 (0.62, 4.11)	0.408	2.44 (0.99, 6.98)	0.069	1.65 (0.97, 2.81)	0.063
Peritonitis	0.0 (0)	0.0 (0)	0.0 (0)	-	-	-	-	-	-
Blood in stools or bloody diarrhea	0.0 (0)	0.0 (0)	0.0 (0)	-	-	-	-	-	-
Bowel ischaemia	2.5 (1)	0.0 (0)	2.2 (5)	1.02 (0.15, 2.00)	0.988	-	-	-	-
Kidney involvement	50.0 (20)	88.1 (111)	77.2 (179)	**3.27 (1.63, 6.58)**	**<0.001**	**7.82 (3.42, 18.49)**	**<0.001**	**2.39 (1.28, 4.72)**	**0.008**
Hypertension >95th centile (for height)	17.5 (7)	33.3 (42)	19.4 (45)	1.04 (0.45, 2.71)	0.937	2.41 (1.02, 6.45)	0.058	**2.33 (1.37, 3.99)**	**0.002**
Proteinuria >0.3g/24h, >20mg/mmol creatinine	30.0 (12)	84.1 (106)	63.4 (147)	**3.92 (1.92, 8.42)**	**<0.001**	**11.93 (5.29, 28.50)**	**<0.001**	**3.04 (1.75, 5.49)**	**<0.001**
Haematuria ≥ 2+ or 5 rbc/hpf or red blood cell casts	27.5 (11)	79.4 (100)	69.4 (161)	**5.72 (2.77, 12.60)**	**<0.001**	**10.69 (4.78, 25.51)**	**<0.001**	**1.87 (1.10, 3.26)**	**0.024**
GFR 50-80ml/min/1.73m^2	10.0 (4)	3.2 (4)	5.2 (12)	0.48 (0.15, 1.79)	0.224	0.33 (0.07, 1.48)	0.134	0.69 (0.18, 2.13)	0.545
GFR 15-49ml/min/1.73m^2	5.0 (2)	19.8 (25)	8.2 (19)	1.62 (0.44, 10.49)	0.526	**4.95 (1.36, 31.91)**	**0.036**	**3.05 (1.55, 6.11)**	**0.001**
GFR <15ml/min/1.73m^2	2.5 (1)	15.9 (20)	6.0 (14)	2.54 (0.49, 46.82)	0.374	7.27 (1.42, 133.14)	0.058	**2.86 (1.34, 6.23)**	**0.007**
Rise in creatinine >10% or Creatinine clearance (GFR) fall >25%	0.0 (0)	0.8 (1)	0.4 (1)	-	-	-	-	1.06 (0.04, 29.32)	0.967
Nervous system involvement	22.5 (9)	20.6 (26)	15.9 (37)	0.64 (0.29, 1.53)	0.288	0.99 (0.42, 2.46)	0.978	1.55 (0.85, 2.78)	0.145
Headache	17.5 (7)	13.5 (17)	9.1 (21)	0.46 (0.19, 1.25)	0.106	0.76 (0.30, 2.14)	0.585	1.65 (0.80, 3.38)	0.172
Meningitis/encephalitis	0.0 (0)	0.0 (0)	0.0 (0)	-	-	-	-	-	-
Organic confusion/cognitive dysfunction	2.5 (1)	1.6 (2)	0.4 (1)	0.13 (0.01, 3.54)	0.165	0.42 (0.03, 10.58)	0.527	3.10 (0.19, 80.69)	0.422
Seizures (not hypertensive)	2.5 (1)	4.8 (6)	2.2 (5)	0.83 (0.13, 16.22)	0.868	1.89 (0.30, 36.79)	0.566	2.28 (0.62, 8.62)	0.210
Stroke	2.5 (1)	0.0 (0)	0.0 (0)	-	-	-	-	-	-
Cord lesion	0.0 (0)	0.0 (0)	0.0 (0)	-	-	-	-	-	-
Cranial nerve palsy	0.0 (0)	2.4 (3)	4.3 (10)	-	-	-	-	0.61 (0.13, 2.12)	0.472
Sensory peripheral neuropathy	2.5 (1)	1.6 (2)	1.3 (3)	0.50 (0.06, 10.84)	0.572	0.94 (0.08, 21.59)	0.964	1.87 (0.23, 12.15)	0.509
Motor mononeuritis multiplex	0.0 (0)	0.8 (1)	0.9 (2)	-	-	-	-	0.98 (0.04, 10.62)	0.990

(-) indicates unreliable statistical results due to an insufficient sample size in one or both of the groups being compared.

Supplementary Table 5. Kidney function over the first 12 months in PR3-ANCA and MPO-ANCA positive pediatric-onset AAV.

	Mean eGFR ± SD	Mean Difference (95 % CI)	*P*-value
**(A) MPO-ANCA**			
**(I) Stage 1-2**			
6-months vs. diagnosis	105.9 ± 29.1 *vs.* 111.0 ± 23.5	-5.12 (-19.60, 9.35)	0.488
12-months vs. diagnosis	108.3 ± 25.3 *vs.* 111.0 ± 23.5	-2.50 (-17.30, 12.30)	0.741
12-months vs. 6-months	108.3 ± 25.3 *vs.* 105.9 ± 29.1	2.62 (-12.20, 17.43)	0.729
**(II) Stage 3**			
6-months vs. diagnosis	66.1 ± 21.0 *vs.* 44.3 ± 11.7	26.60 (-1.08, 54.40)	0.060
12-months vs. diagnosis	74.8 ± 32.3 *vs.* 44.3 ± 11.7	30.50 (5.47, 55.60)	**0.017**
12-months vs. 6-months	74.8 ± 32.3 *vs.* 66.1 ± 21.0	3.89 (-23.84, 31.60)	0.784
**(III) Stage 4-5**			
6-months vs. diagnosis	38.2 ± 32.4 *vs.* 13.0 ± 7.1	23.50 (12.57, 34.40)	**<0.001**
12-months vs. diagnosis	44.6 ± 39.4 *vs.* 13.0 ± 7.1	28.76 (17.60, 39.90)	**<0.001**
12-months vs. 6-months	44.6 ± 39.4 *vs.* 38.2 ± 32.4	5.29 (-6.34, 16.90)	0.373
**(B) PR3-ANCA**			
**(I) Stage 1-2**			
6-months vs. diagnosis	95.6 ± 27.6 *vs.* 109.9 ± 25.3	-14.97 (-22.10, -7.82)	**<0.001**
12-months vs. diagnosis	91.7 ± 27.6 *vs.* 109.9 ± 25.3	-19.20 (-26.40, -11.90)	**<0.001**
12-months vs. 6-months	91.7 ± 27.6 *vs.* 95.6 ± 27.6	-4.19 (-11.80, 3.45)	0.283
**(II) Stage 3**			
6-months vs. diagnosis	61.2 ± 33.7 *vs.* 42.1 ± 7.6	20.00 (5.03, 34.90)	**0.009**
12-months vs. diagnosis	68.7 ± 35.9 *vs.* 42.1 ± 7.6	26.20 (10.18, 42.20)	**0.001**
12-months vs. 6-months	68.7 ± 35.9 *vs.* 61.2 ± 33.7	6.22 (-9.95, 22.40)	0.451
**(III) Stage 4-5**			
6-months vs. diagnosis	25.0 ± 19.2 *vs.* 11.8 ± 7.1	12.30 (-2.27, 26.80)	0.098
12-months vs. diagnosis	20.5 ± 19.3 *vs.* 11.8 ± 7.1	5.76 (-10.70, 22.20)	0.493
12-months vs. 6-months	20.5 ± 19.3 *vs.* 25.0 ± 19.2	-6.49 (-23.13, 10.10)	0.444
